# Correlates of disease-specific knowledge among patients with chronic hepatitis B or hepatitis C infection in India

**DOI:** 10.1007/s12072-016-9728-3

**Published:** 2016-05-04

**Authors:** Aracely Tamayo, Samir R. Shah, Shobna Bhatia, Abhijit Chowdhury, Padaki N. Rao, Phillip Dinh, Steven J. Knox, Anuj Gaggar, G. Mani Subramanian, Viswanathan G. Mohan, Ajit Sood, Rajiv Mehta, Shiv K. Sarin

**Affiliations:** 1 University of California Berkeley School of Public Health, Berkeley, CA USA; 2 Jaslok Hospital and Research Centre and Breach Candy Hospital, Mumbai, India; 3 Seth GS Medical College and KEM Hospital, Mumbai, India; 4 Institute of Post Graduate Medical Education and Research, Kolkata, India; 5 Asian Institute of Gastroenterology, Hyderabad, India; 6 Gilead Sciences, Inc., Foster City, CA USA; 7 VGM Hospital, Coimbatore, Tamil Nadu India; 8 Dayanand Medical College and Hospital, Ludhiana, India; 9 Liver Clinic, Surat, India; 10 Institute of Liver and Biliary Sciences, New Delhi, India

**Keywords:** Hepatitis B virus, Hepatitis C virus, Disease knowledge, India

## Abstract

**Background:**

Patient knowledge about chronic diseases increases health-promoting behaviors and improves clinical outcomes. We assessed this association for patients with chronic viral hepatitis.

**Methods:**

Untreated patients chronically infected with HBV (*n* = 500) or HCV (*n* = 500) were enrolled at 19 centers across India. A survey, adapted from the US CDC National Health and Nutrition Examination Survey (NHANES) questionnaire, was administered at a single visit to assess HBV/HCV knowledge, community disease awareness, treatment quality, and healthcare barriers. We developed the India Hepatitis Knowledge Index (IHKI), where a higher IHKI score (range 0–10) indicates increased hepatitis knowledge. Multivariate regression models evaluated demographic and disease factors.

**Results:**

The overall mean IHKI score was 5.6 out of 10, with higher scores among patients with HBV (5.9) than HCV (5.3); *p* < 0.001. In HBV patients lower IHKI was associated with shorter disease duration, government clinic attendance (*p* < 0.0001), fewer personal experiences with HBV (*p* < 0.0001), and residing in northern India. Among HCV patients, lower IHKI was associated with shorter disease duration, community (*p* < 0.0001) and government clinic attendance (*p* < 0.0001), and fewer personal experiences with HCV (*p* < 0.0001). Among HBV patients, IHKI was independently associated with disease severity as assessed by MELD score, albumin, and APRI. This association was strongest for HBV patients with elevated ALT and HBV DNA >2000 IU/ml. Among HCV patients, IHKI results had no significant associations with disease severity.

**Conclusions:**

The association of IHKI with disease underscores the need to understand connections between hepatitis knowledge and progression and may guide efforts to address patient education and awareness of chronic viral hepatitis in India.

**Electronic supplementary material:**

The online version of this article (doi:10.1007/s12072-016-9728-3) contains supplementary material, which is available to authorized users.

## Background

HBV and HCV infections continue to be global health concerns, affecting over 500 million people worldwide [1]. India remains a country with a high burden of viral hepatitis. It is estimated that the prevalence of HCV is ~0.5–1.0 % and ~2–8 % for HBV [2–6]. While infection rates are high, engagement with the healthcare system is limited in part by access to healthcare systems and individual knowledge of disease transmission, natural history, and treatment options [7, 8]. With the availability of a preventative vaccine for HBV and effective therapies for HBV and HCV, improving interactions with healthcare systems may improve overall health for those infected with HBV and HCV.

Hepatitis knowledge has been demonstrated to be associated with disease management and outcomes, with improved management and outcomes among those with increased knowledge of the disease [8, 9]. Further, as knowledge of transmission improves, rates of disease screening and incidence have been shown to decrease [10]. There is a lack of data, however, assessing the overall knowledge of disease for viral hepatitis and evaluating associations with clinical parameters. Assessing the current status of disease knowledge is important for demonstrating knowledge gaps that ultimately will be useful for designing specific public health interventions.

In this study, we assess disease knowledge of HBV and HCV in India using a series of questions administered to 500 HBV and 500 HCV patients throughout India. The goals of this study are to (1) characterize and validate answers to a series of questions adapted from the NHANES questionnaire, (2) identify differences in knowledge awareness of disease between HBV- and HCV-infected patients, and (3) identify associations with clinical parameters.

## Methods

### Study population

A total of 500 HBV and 500 HCV patients were recruited from 19 centers geographically distributed across India. Practice types included community clinics, government hospitals, government assistance programs, and private practices. At a single study visit, a survey was administered and clinical laboratory tests were performed. The surveys collected information on participants’ sociodemographic characteristics, hepatitis-related knowledge, experiences, and behaviors and were subsequently linked to participants’ laboratory and clinical measures. This study was approved by the Institutional Review Boards governing the participating centers.

## Description of study variables

### India Hepatitis Knowledge Index

We used ten hepatitis survey questions adapted from the NHANES follow-up questionnaire for hepatitis C to create the India Hepatitis Knowledge Index (IHKI) [11]. These ten questions assessed patient knowledge of hepatitis disease transmission, natural history, treatment, and prevention options. HBV and HCV patients were only asked about knowledge regarding their specific hepatitis type.

We conducted a factor analysis by hepatitis type to assess whether items on the knowledge scale hold together as one or more constructs or measures of knowledge. Only one distinct factor of knowledge was identified in factor analysis. For each hepatitis type, a Cronbach’s alpha (*α*) for the reliability of our ten scale items to assess the adequacy of scale reliability was performed. We created IHKI by hepatitis B or C patients. The index score was the number of correct questions (index range 0–10).

### Clinical laboratory tests/values

At the clinic visit, clinical laboratory assessments were made including AST, ALT, albumin, bilirubin (total and direct), INR for prothrombin time, GGT, and creatinine. Viral factors evaluated were HCV RNA and HCV genotyping (for those with HCV infection) and HBV DNA, HBeAg, and anti-HBeAg (for those with HBV infection). MELD and APRI calculations were performed for all patients.

For sensitivity analyses, we categorized patients into high ALT or low ALT using separate cutoffs for males and females. For males, high ALT was defined as ALT ≥90 and low ALT was defined as ALT <90; for females, high ALT was defined as ALT ≥57 and low ALT was defined as ALT <57. We also categorized HBV patients into high and low viral counts (high viral load ≥2000 IU/ml, low viral load <2000 IU/ml).

### Covariates

We included age, sex, duration of infection, type of healthcare practice, geographic region of India, and hepatitis experience as covariates in the analysis. All covariates were collected from the patient survey. Age was measured continuously. Duration of hepatitis was defined as <1 year, 2–5 years, and >5 years. Patients were asked at what type of healthcare practice they received most of their regular care (private practice, community clinic, government hospital/program). The location of participating centers was classified into four distinct regions of India (i.e., north, south, west, and east).

A summary measure of personal experiences with hepatitis was calculated from three survey questions and used to capture previous exposure to hepatitis as a possible predictor of knowledge and clinical outcomes. Personal experience with hepatitis was calculated from questions asking whether the respondent had family members with hepatitis, whether a family member had died from hepatitis, and whether the respondent lived in a community that spoke openly about hepatitis (yes/no). One point was given for every question where the patient answered yes (response range 0–3). The options “refused to answer” and “don’t know” were available for all survey questions.

### Statistical analysis

We assessed associations of hepatitis type, clinical values, and knowledge scores by patient sociodemographic factors using chi-square tests and one-way analysis of variance tests appropriate for our categorical and continuous measures of variables, respectively. We used Pearson correlations to examine associations between continuous sociodemographic characteristics and knowledge scores and median regression to examine associations between laboratory medians and sociodemographic characteristics.

Linear and logistic regressions were used to examine adjusted associations between sociodemographic characteristics and knowledge scores and between knowledge scores and clinical outcomes measured continuously and categorically. Age was centered in our models. Multivariate models for knowledge included age, sex, duration of infection, healthcare clinic type, and personal experience with hepatitis. Models for clinical outcomes included the hepatitis knowledge score.

In our sensitivity analysis we assessed how associations between knowledge and clinical outcomes varied for individuals with low and high ALT and for HBV patients with low and high levels of HBV DNA. All analyses were performed using STATA/SE 13.1.

## Results

Among our sample of 1000 respondents, 1 (<0.1 %) person had missing values for the knowledge score and was excluded from the analysis. In the analytic sample, overall the mean (SD) age was 41 (13) years, 72 % were male, and 75 % were Hindu, 6 % Muslim, 8 % Sikh, 9 % Christian, and 1 % some other religion (Table 1). Over half of respondents reported hepatitis duration of less than 1 year, 28 % reported 2–5 years, 10 % reported having duration of more than 5 years, and 12 % refused to answer or did not know their duration of infection. Most respondents received primary care at private practices (61 %), community clinics (7 %), and government program (28 %), and 12 % refused to report or did not know the type of practice where they received care. Comparing HBV and HCV patients, those with HCV tended to be older (45 versus 37 years), more were females (34.4 versus 21.4 %), and more HBV patients reported a family history of hepatitis (24 versus 14 %). For both HBV and HCV patients the majority of patients had elevated ALT levels. Compared to HBV patients the HCV patients had laboratory values consistent with active or more advanced disease, including ALT (73 versus 50 U/l), albumin (3.8 versus 4.3 g/dl), and APRI (1.4 versus 0.4).

**Table 1:** Sociodemographic characteristics of study respondents

Sociodemographic characteristics	Hepatitis type			
	Total study sample	HBV	HCV	*p* values*
All sample	999	499	500	
Mean age in years (SD)	41 (13)	37 (12)	45 (13)	<0.001
Sex				
Female	278 (28 %)	106 (21 %)	172 (34 %)	<0.001
Male	721 (72 %)	393 (79 %)	328 (66 %)	
Religion				
Hindu	754 (75 %)	405 (81 %)	349 (70 %)	<0.001
Muslim	64 (6 %)	38 (8 %)	26 (5 %)	
Sikh	81 (8 %)	24 (5 %)	57 (11 %)	
Christian	93 (9 %)	28 (6 %)	65 (13 %)	
Other	7 (1 %)	4 (1 %)	3 (1 %)	
Duration of hepatitis^a^				
Less than 1 year	505 (51 %)	259 (52 %)	246 (49 %)	0.365
2–5 years	275 (28 %)	125 (25 %)	150 (30 %)	
>5 years	100 (10 %)	53 (11 %)	47 (9 %)	
Refused/do not know	119 (12 %)	62 (12 %)	57 (11 %)	
Healthcare practice type				
Private practice	606 (61 %)	303 (61 %)	303 (61 %)	0.686
Community clinic	72 (7 %)	36 (7 %)	36 (7 %)	
Government program	282 (28 %)	137 (27 %)	145 (29 %)	
Refused/DK	39 (4 %)	23 (5 %)	16 (3 %)	
Geographic region of India				
North	250 (25 %)	125 (25 %)	125 (25 %)	1.000
South	249 (25 %)	124 (25 %)	125 (25 %)	
West	250 (25 %)	125 (25 %)	125 (25 %)	
East	250 (25 %)	125 (25 %)	125 (25 %)	
Family history of hepatitis^a^				
Yes	194 (19 %)	122 (24 %)	72 (14 %)	<0.001
No/Ref/DK	805 (81 %)	377 (76 %)	428 (86 %)	
Family death due to hepatitis^a^				
Yes	82 (8 %)	49 (10 %)	33 (7 %)	0.064
No/Ref/DK	917 (92 %)	450 (90 %)	467 (93 %)	
Community awareness of hepatitis^a^				
Yes	303 (30 %)	147 (29 %)	156 (31 %)	0.549
No/Ref/DK	696 (70 %)	352 (71 %)	344 (69 %)	
Clinical laboratory values, median (IQR)				
ALT, U/l		50 (39–72)	73 (50–112)	<0.001
Albumin, g/dl		4.3 (3.9–4.5)	3.8 (3.2–4.3)	<0.001
Total bilirubin, g/dl		0.6 (0.4–1.0)	0.8 (0.5–1.3)	<0.001
Direct bilirubin, mg/dl		0.1 (0.1–0.2)	0.2 (0.1–0.5)	<0.001
APRI		0.4 (0.3–1.0)	1.4 (0.6–3.1)	<0.001
MELD		7 (6–9)	8 (7–11)	<0.001

* *p* values were calculated using chi-square tests of association for categorical sociodemographic characteristics and analysis of variance for continuous sociodemographic characteristics

^a^For HBV respondents, questions were asked in reference to HBV; for HCV respondents, questions were asked in reference to HCV

Correct responses to the ten knowledge questions are shown in Table 2. Correct responses to questions ranged from 35 to 73 % for patients with HBV and 32–78 % for HCV. For most questions, there were no statistically significant differences in correct responses according to HBV versus HCV infection. Notably there were two questions regarding transmission (i.e., if sex with an infected partner is a risk factor for infection or whether hepatitis can be transmitted from mother to child through birth) and one question concerning the availability of a vaccine (all *p* < 0.0001) in which more HBV patients answered the questions correctly. We summed the number of correctly answered knowledge questions to create a knowledge index. For each hepatitis type (i.e., HBV versus HCV), we found the adequacy of index reliability (HBV: *α* = 0.81; HCV *α* = 0.75), and the validity of the constructed knowledge composite score was determined by performing a factor analysis to identify the number of underlying factors measured by the knowledge questions. For both HBV and HCV, this analysis demonstrated a one-factor solution for both HBV and HCV patients (Supplemental table 1). We also evaluated the internal consistency of the index using Cronbach’s alpha, which demonstrated a high index of reliability (HBV: *α* = 0.81; HCV *α* = 0.75). The IHKI score was set as the number of correct questions answered by an individual. The overall mean IHKI score for the entire population was 5.6 out of 10 (SD 2.8). Overall, HBV patients had higher knowledge scores than HCV patients (HBV scores 5.9 than HCV scores 5.3; *p* < 0.001). Questions that appeared to contribute the most to differences in knowledge were those associated with transmission and vaccination.

**Table 2:** Hepatitis virus knowledge scale results

Hepatitis knowledge question^a^	HBV correct response (*n* = 499) *N* (%)	HCV correct response (*n* = 500) *N* (%)	*p* value*
Can hepatitis B/C patients transmit the disease to others?	295 (59 %)	267 (53 %)	0.069
Is having sex with someone who suffers from hepatitis B/C a risk factor for getting infected with hepatitis B/C?	268 (54 %)	209 (42 %)	<0.001
You can get hepatitis B/C by being born to a mother who suffers from hepatitis B/C when she gave birth	252 (51 %)	187 (37 %)	<0.001
You can get hepatitis B/C by being pricked with a needle or sharp instrument or injecting illegal drugs with a needle that has hepatitis B/C-infected blood on it	351 (70 %)	362 (72 %)	0.472
You can get hepatitis B/C by receiving a blood transfusion from a hepatitis B/C-infected donor	365 (73 %)	389 (78 %)	0.087
If someone is infected with hepatitis B/C virus they will most probably carry the virus all their lives	176 (35 %)	172 (34 %)	0.773
Infection with the hepatitis B/C virus can cause the liver to stop functioning	252 (51 %)	250 (50 %)	0.874
Someone suffering from hepatitis B/C can look and feel fine	307 (62 %)	305 (61 %)	0.865
Is hepatitis B/C a treatable disease?	345 (69 %)	352 (70 %)	0.664
Is there a vaccine available for hepatitis B/C?	351 (70 %)	162 (32 %)	<0.001

* *p* values were calculated using chi-square tests of association

^a^For HBV respondents, questions were asked in reference to HBV; for HCV respondents, questions were asked in reference to HCV

The average knowledge scores by sociodemographic characteristics are shown in Table 3. Males and females with HBV had similar knowledge scores; however, for HCV, females had lower knowledge scores than males. Overall, respondents with a longer duration of infection and more personal family or community awareness of hepatitis had higher knowledge scores. In the overall population and for the HBV and HCV groups, there was a trend toward increased knowledge with a longer duration of infection, attending private practice, a family history of hepatitis and hepatitis-related death, and community awareness of disease. While not a predictor for either HBV or HCV, the overall group of patients showed a weak trend toward increased knowledge with lower age. Groups that performed the highest in the overall knowledge score included HBV patients with >5 years of disease duration and HBV patients with a family death due to hepatitis.

**Table 3:** Sociodemographic characteristic and hepatitis knowledge score

Sociodemographic characteristics	Overall knowledge score	*p* value	Hepatitis type			
			HBV knowledge score	*p* value	HCV knowledge score	*p* value
All sample	999		499		500	
Age (correlation)	−0.08	0.009	−0.06	0.217	0.2166	0.321
Sex						
Female	5.4	0.060	6.0	0.916	5.0	0.044
Male	5.7		6.0		5.5	
Religion						
Hindu	5.7	0.119	6.0	0.1660	5.2	0.13
Muslim	5.1		5.5		4.5	
Sikh	5.1		4.6		5.4	
Christian	6.0		6.2		5.9	
Other	6.4		6.3		6.7	
Duration of hepatitis^a^						
Less than 1 year	5.4	<0.001	5.7	<0.001	5.1	<0.001
2–5 years	6.2		6.6		5.9	
>5 years	6.6		7		6.0	
Refused/DK	4.3		4.4		4.1	
Healthcare practice type						
Private practice	6.1	<0.001	6.4	<0.001	5.7	<0.001
Community clinic	5.0		5.6		4.4	
Government program	5.0		5.3		4.8	
Refused/missing	4.1		4.2		3.9	
Geographic region of India						
North	5.5	0.516	5.6	0.178	5.4	0.012
South	5.8		6.1		5.4	
West	5.5		6.3		4.7	
East	5.7		5.7		5.7	
Family history of hepatitis^a^						
Yes	6.5	<0.001	6.9	<0.001	6.0	0.018
No/Ref/DK	5.4		5.6		5.2	
Family death due to hepatitis^a^						
Yes	6.7	<0.001	7.0	<0.001	6.2	0.035
No/Ref/DK	5.5		5.8		5.2	
Community awareness of hepatitis^a^						
Yes	6.2	<0.001	6.7	<0.001	5.8	<0.001
No/Ref/DK	5.4		5.6		5.1	

* *p* values were calculated using analysis of variance for categorical sociodemographic characteristics and Pearson correlations for continuous variables

^a^For HBV respondents, questions were asked in reference to HBV; for HCV respondents, questions were asked in reference to HCV

Multivariate analysis did not show age or gender to be associated with knowledge scores for hepatitis B or C (Table 4). For both HBV and HCV the duration of disease, type of healthcare practice, geographic regions in India, and personal experience with hepatitis were all associated with the mean knowledge score. Longer duration of infection, 2–5 years (for HBV, coefficient: 1.0 CI: 0.42, 1.6 and for HCV, coefficient: 0.8 CI: 0.28, 1.31) or more than 5 years (for HBV, coefficient: 1.1 CI: 0.29, 1.92 and for HCV, coefficient: 1.0 CI: 0.22, 1.79), was related to higher mean knowledge scores compared to respondents with less than 1 year since the time of diagnosis. Attending a community clinic (for HCV, coefficient: −1.43 CI: −2.32, −0.53) and government clinics (for HBV, coefficient: −0.085 CI: −1.44, −0.27 and for HCV, coefficient: −0.84 CI: −1.38, −0.31) was also associated with lower mean knowledge scores compared to those attending private practices. Increased personal experiences with hepatitis also were positively associated with increases in the knowledge score, and every additional experience with disease (0–3) increased the knowledge score by 0.82 (CI 0.51, 1.13). In adjusted models, patients who refused to answer or did not know the duration of infection or type of primary care clinic had the lowest knowledge scores compared to those with <1 year disease duration and those visiting private practice clinics.

**Table 4:** Multivariate regression for predictors of knowledge scores

Factors	HBV					HCV				
	*b*	SE	*p* value	LCI	UCI	*b*	SE	*p* value	LCI	UCI
Age	−0.01	0.01	0.21	−0.03	0.01	−0.02	0.01	0.10	−0.03	0.00
Sex										
Male	REF					REF				
Female	−0.07	0.30	0.82	−0.66	0.52	−0.46	0.24	0.06	−0.93	0.01
Duration of hepatitis^a^										
Less than 1 year	REF					REF				
2–5 years	1.01	0.30	0.00	0.42	1.60	0.80	0.26	0.00	0.28	1.31
>5 years	1.10	0.41	0.01	0.29	1.92	1.00	0.40	0.01	0.22	1.79
Refused/DK	−0.90	0.40	0.02	−1.68	−0.12	−0.83	0.37	0.02	−1.55	−0.11
Healthcare practice type										
Private practice	REF					REF				
Community clinic	−0.42	0.49	0.39	−1.38	0.54	−1.43	0.46	0.00	−2.32	−0.53
Government program	−0.85	0.30	0.00	−1.44	−0.27	−0.84	0.27	0.00	−1.38	−0.31
Refused/missing	−1.76	0.59	0.00	−2.92	−0.60	−1.60	0.64	0.01	−2.85	−0.35
Geographic region of India										
North	REF					REF				
South	0.58	0.36	0.11	−0.13	1.29	0.15	0.35	0.67	−0.53	0.83
West	0.69	0.35	0.05	0.00	1.39	−0.62	0.34	0.06	−1.28	0.03
East	0.18	0.36	0.63	−0.53	0.88	0.39	0.33	0.24	−0.26	1.04
Personal experiences with hepatitis^a,b^	0.82	0.16	0.00	0.51	1.13	0.54	0.16	0.00	0.22	0.86
_Cons	5.10	0.35	0.00	4.42	5.79	5.43	0.34	0.00	4.77	6.09

^a^For HBV respondents, questions were asked in reference to HBV; for HCV respondents, questions were asked in reference to HCV

^b^Personal experiences were derived from questions asking if the respondent had family members with hepatitis, if any family member had died from hepatitis, and whether they lived in a community that spoke about hepatitis (yes/no). One point was given for every question where the patient answered yes (response range: 0–3)

### Association with clinical parameters

In adjusted analysis, we found that increased knowledge was related to better clinical outcomes for hepatitis B patients (Table 5). An increase in the IHKI score was related to lower MELD and APRI scores and lower INR, and bilirubin (total and direct) while associated with higher albumin levels. For patients with hepatitis C, no significant associations were seen although a trend toward lower MELD and higher albumin was seen with improved knowledge. In sensitivity analysis, we found the associations for hepatitis B to be strengthened for high HBV DNA and elevated ALT, a subgroup of hepatitis B patients who may have increased liver damage or more advanced liver disease (Supplemental table 2).

**Table 5:** Associations from adjusted linear or logistic regression of hepatitis knowledge and clinical outcomes by hepatitis type

Continuous clinical outcomes	HBV					HCV				
	*b*	SE	*p* value	LCI	UCI	*b*	SE	*p* value	LCI	UCI
MELD	−0.21	0.06	0.00	−0.33	−0.08	−0.16	0.09	0.08	−0.33	0.02
Albumin	0.02	0.01	0.01	0.01	0.04	0.02	0.01	0.07	0.00	0.04
PT/INR	−0.01	0.01	0.01	−0.02	0.00	0.00	0.00	0.87	−0.01	0.01
Total bilirubin	−0.21	0.06	0.00	−0.33	−0.08	−0.01	0.03	0.75	−0.07	0.05
Direct bilirubin	−0.13	0.04	0.00	−0.22	−0.05	−0.01	0.02	0.64	−0.04	0.02
ALT	−3.58	3.91	0.36	−11.25	4.09	1.91	1.25	0.13	−0.54	4.36

Dichotomized clinical outcomes	HBV					HCV				
	OR	SE	*p* value	LCI	UCI	OR	SE	*p* value	LCI	UCI
APRI	0.84	0.04	0.00	0.76	0.93	1.01	0.04	0.87	0.93	1.09
High ALT	0.95	0.04	0.20	0.87	1.03	1.01	0.04	0.84	0.94	1.09

Models adjusted for age, sex, healthcare practice type, region of India, and personal experiences with hepatitis

## Conclusions

In conclusion, our study demonstrates that a reliable scale of knowledge can be created using a simple survey that can be administered across diverse regions in India. Our results show that patients with HBV tend to have higher awareness of their disease and specifically have increased knowledge about modes of transmission and the availability of a vaccine as compared with patients with HCV. As might be expected, factors related to improved disease awareness included longer duration of infection and personal experience with hepatitis such as a family history of or family death from hepatitis. We did note that there was a trend toward an association of lower age and improved knowledge for the overall population, which may reflect an increase in knowledge in younger populations as a result of improved access to information. Of note, in the overall population religion, gender, and region of India were not associated with the knowledge score on univariate analysis. Finally, receiving care in a private practice setting was associated with higher knowledge scores for both HBV and HCV, which may be tied to socioeconomic status (SES). As SES was not assessed in this study, the reason for these improved scores among patients being seen in private practice cannot be definitively assessed.

Somewhat surprisingly, even with a limited set of questions evaluating knowledge, an association was seen with important clinical parameters including MELD, APRI, albumin, and bilirubin for HBV patients. This association may be interpreted to mean that individuals with lower knowledge about HBV tend to present in healthcare settings later and with more advanced disease. This is strengthened by the finding of an augmented effect among HBV patients with elevated ALT and HBV DNA. Given this is a single-visit, cross-sectional study, causality cannot be determined. These associations were more apparent for HBV, whereas only trends were noted for HCV. The reason for the lack of statistical significance among HCV patients may reflect higher values and variability in clinical laboratory results among HCV patients due to more advanced liver disease than in HBV patients or due to differences in sociodemographic predictors for contracting HCV versus HBV infection.

Despite the relatively large sample size and broad geographic distribution of the survey, there are several limitations of this study. While multiple centers were used for the study, sites were chosen based on feasibility rather than on a population-based sample, thereby limiting the generalizability of the findings to India and other populations. While broad concepts could be assessed with this simple survey, we were unable to evaluate more subtle differences in knowledge with only ten questions. This survey was also performed in conjunction with a blood draw, which may also introduce selection bias among patients being seen at these clinics. Finally, we did not collect the SES and educational levels of patients in our study, which may be important possible confounders for the results observed. While SES may be inferred from the practice type in which patients were seen, no definitive assessment of either SES or education was known, and no additional analysis was possible.

The findings of this survey demonstrate the overall low level of knowledge of basic disease transmission and natural history among patients in India and highlights the need for further education concerning HBV and HCV. Further, that lower disease knowledge may be associated with more advanced disease at the time of presentation may imply a tangible benefit of improved education among the population. With effective preventative and therapeutic options for HBV and HCV infection, improving disease knowledge may impact the overall disease burden in India.

## Electronic supplementary material

Below is the link to the electronic supplementary material.

Supplementary material 1 (DOCX 36 kb)
