# Supplementary material for: Correlates of disease-specific knowledge among patients with chronic hepatitis B or hepatitis C infection in India
Source: Hepatol Int. 2016 May 4;10(6):988–95. doi: 10.1007/s12072-016-9728-3 (PMC5083769; doi:10.1007/s12072-016-9728-3)
Supplement: Supplementary file 1 — Supplementary material 1 (DOCX 36 kb) [file 12072_2016_9728_MOESM1_ESM.docx]

Supplementary materials to:

**Correlates of disease-specific knowledge among patients with chronic hepatitis B or hepatitis C infection in India**

Aracely Tamayo, Samir R Shah, Shobna Bhatia, Abhijit Chowdhury, Padaki N Rao, Phillip Dinh, Steven J Knox, Anuj Gaggar, G Mani Subramanian, Viswanathan G Mohan, Ajit Sood, Rajiv Mehta, Shiv K Sarin

**Corresponding author:**

Aracely Tamayo

University of California Berkeley School of Public Health, Berkeley, CA, USA

[tamayo@berkeley.edu](mailto:tamayo@berkeley.edu)

**Supplemental Table 1.** Adjusted Linear Regression of Hepatitis Knowledge and Clinical Outcomes by high/low ALT levels for HBV and HCV

|  | **HBV** | | | | | | | | | |
| --- | --- | --- | --- | --- | --- | --- | --- | --- | --- | --- |
|  | **HBV High Alt** | | | | | **HBV Low Alt** | | | | |
| **Continuous Clinical Outcomes** | **b** | **SE** | **p-value** | **LCI** | **UCI** | **b** | **SE** | **p-value** | **LCI** | **UCI** |
|  |  |  |  |  |  |  |  |  |  |  |
| MELD | -0.65 | 0.26 | 0.01 | -1.16 | -0.13 | -0.08 | 0.05 | 0.07 | -0.17 | 0.01 |
| Albumin | 0.04 | 0.03 | 0.15 | -0.01 | 0.09 | 0.02 | 0.01 | 0.06 | 0.00 | 0.04 |
| APRI | 0.83 | 0.07 | 0.03 | 0.70 | 0.98 | 0.75 | 0.08 | 0.01 | 0.61 | 0.93 |
| PT/INR | -0.06 | 0.03 | 0.02 | -0.11 | -0.01 | 0.00 | 0.00 | 0.07 | -0.01 | 0.00 |
| Total Bilirubin | -0.81 | 0.32 | 0.01 | -1.44 | -0.17 | -0.03 | 0.01 | 0.03 | -0.05 | 0.00 |
| Direct Bilirubin | -0.53 | 0.22 | 0.02 | -0.96 | -0.10 | -0.01 | 0.01 | 0.01 | -0.03 | 0.00 |
|  |  |  |  |  |  |  |  |  |  |  |
| **Dichotomized Clinical Outcomes** | **OR** | **SE** | **p-value** | **LCI** | **UCI** | **OR** | **SE** | **p-value** | **LCI** | **UCI** |
| APRI | 0.75 | 0.08 | 0.01 | 0.61 | 0.93 | 0.83 | 0.07 | 0.03 | 0.70 | 0.98 |
|  |  |  |  |  |  |  |  |  |  |  |
|  | **HCV** | | | | | | | | | |
| **HCV** | **HCV High Alt** | | | | | **HCV Low Alt** | | | | |
| **Clinical Outcomes** | **b** | **SE** | **p-value** | **LCI** | **UCI** | **b** | **SE** | **p-value** | **LCI** | **UCI** |
|  |  |  |  |  |  |  |  |  |  |  |
| MELD | -0.09 | 0.10 | 0.40 | -0.29 | 0.12 | -0.23 | 0.15 | 0.12 | -0.52 | 0.06 |
| Albumin | 0.01 | 0.01 | 0.30 | -0.01 | 0.04 | 0.03 | 0.02 | 0.10 | -0.01 | 0.06 |
| PT/INR | 0.01 | 0.00 | 0.10 | 0.00 | 0.02 | -0.01 | 0.01 | 0.11 | -0.02 | 0.00 |
| Total Bilirubin | -0.03 | 0.04 | 0.53 | -0.12 | 0.06 | 0.01 | 0.04 | 0.88 | -0.07 | 0.08 |
| Direct Bilirubin | -0.02 | 0.02 | 0.30 | -0.07 | 0.02 | 0.01 | 0.02 | 0.70 | -0.04 | 0.06 |
|  |  |  |  |  |  |  |  |  |  |  |
| **Dichotomized Clinical Outcomes** | **OR** | **SE** | **p-value** | **LCI** | **UCI** | **OR** | **SE** | **p-value** | **LCI** | **UCI** |
| APRI | 1.07 | 0.06 | 0.23 | 0.96 | 1.20 | 0.96 | 0.06 | 0.50 | 0.85 | 1.08 |
|  |  |  |  |  |  |  |  |  |  |  |
| Models adjusted for age, sex, health care practice type, region of India, and personal experiences with hepatitis. | | | | | | | | | | |

**Supplemental Table 2.** Adjusted Linear and Logistic Regression of Hepatitis Knowledge and Clinical outcomes by high/low DNA levels for HBV

|  | **HBV High HBV DNA** | | | | | **HBV Low HBV DNA** | | | | |
| --- | --- | --- | --- | --- | --- | --- | --- | --- | --- | --- |
| **Clinical Outcomes** | **b** | **SE** | **p-value** | **LCI** | **UCI** | **b** | **SE** | **p-value** | **LCI** | **UCI** |
|  |  |  |  |  |  |  |  |  |  |  |
| MELD | -0.35 | 0.13 | 0.01 | -0.60 | -0.10 | -0.18 | 0.10 | 0.08 | -0.37 | 0.02 |
| Albumin | 0.05 | 0.02 | 0.02 | 0.01 | 0.08 | 0.00 | 0.01 | 0.69 | -0.02 | 0.00 |
| PT/INR | -0.02 | 0.01 | 0.01 | -0.03 | 0.00 | -0.02 | 0.01 | 0.05 | -0.05 | 0.00 |
| Total Bilirubin | -0.40 | 0.15 | 0.01 | -0.70 | -0.09 | -0.21 | 0.08 | 0.01 | -0.37 | -0.04 |
| Direct Bilirubin | -0.23 | 0.10 | 0.02 | -0.43 | -0.03 | -0.15 | 0.06 | 0.02 | -0.27 | -0.03 |
|  |  |  |  |  |  |  |  |  |  |  |
|  | **OR** | **SE** | **p-value** | **LCI** | **UCI** | **OR** | **SE** | **p-value** | **LCI** | **UCI** |
| APRI | 0.82 | 0.06 | 0.01 | 0.70 | 0.95 | 0.75 | 0.11 | 0.04 | 0.57 | 0.99 |
| High ALT | 1.00 | 0.06 | 0.94 | 0.88 | 1.13 | 0.85 | 0.07 | 0.05 | 0.72 | 1.00 |
|  |  |  |  |  |  |  |  |  |  |  |
